# Newborn Screening for Cystic Fibrosis and Its Incidence at a Tertiary Care Center in Saudi Arabia: A Retrospective Study

**DOI:** 10.7759/cureus.114630

**Published:** 2026-08-16

**Authors:** Hamza M Kelabi, Fahad J Alharbi, Adel Alharbi, Abdullah S Alshamrani, Mansour Alqwaiee, Ayed M Alenazi, Albandari S Alsaban, Khaled A Baqias

**Affiliations:** 1 Department of Pediatric Pulmonary Medicine, Prince Sultan Military Medical City, Riyadh, SAU; 2 Department of Laboratory Medicine, Prince Sultan Military Medical City, Riyadh, SAU

**Keywords:** consanguinity, cystic fibrosis, immune reactive trypsinogen, incidence, newborn screening, saudi arabia, sweat chloride test

## Abstract

Background: Cystic fibrosis (CF) is a life-limiting genetic disorder where early detection via newborn screening (NBS) significantly improves clinical outcomes. Data regarding CF incidence and NBS efficacy in Saudi Arabia remain limited. This single-center pilot study aimed to evaluate the primary diagnostic performance, local incidence, and CFTR genotype distribution using an immunoreactive trypsinogen (IRT)-IRT NBS protocol implemented exclusively at our institution, Prince Sultan Military Medical City (PSMMC), Riyadh.

Methods: A retrospective analysis was conducted on 10,286 newborns screened. between May 2017 and April 2019 using a two-tiered IRT sequence (Day 1 and Day 5). Screen-positive infants underwent confirmatory sweat conductivity analysis and CFTR variant testing.

Results: Three infants were confirmed with CF, yielding a regional incidence of 1:3,429 live births (2.9 per 10,000). Day 5 IRT levels were significantly higher in confirmed CF cases than in non-CF infants (136.07 ± 27.24 vs. 19.44 ± 13.08 ng/mL, p = 0.003) and strongly correlated with sweat conductivity (r = 0.698, p < 0.001). Receiver operating characteristic (ROC) analysis identified an optimal Day 5 IRT cutoff of 114.9 ng/mL (area under the ROC curve (AUC)= 0.999), demonstrating 75.0% sensitivity and 99.8% specificity. Genetic confirmation identified three distinct homozygous variants (c.1416delG, I1234V, and c.1418delG).

Conclusion: This descriptive pilot study conducted at our center demonstrates the feasibility and diagnostic utility of the IRT-IRT screening protocol for early CF detection. To build upon these single-center findings and support the development of a comprehensive national screening strategy, future multi-center studies across Saudi Arabia are recommended.

## Introduction

Cystic fibrosis (CF) is a severe, life-limiting genetic disease that affects an estimated 100,000 people globally. Over the last few decades, advances in therapeutic approaches, interdisciplinary care, and early diagnostic procedures have led to significant improvements in patient outcomes and survival rates [1]. Newborn screening (NBS) programs for CF are well-established public health strategies that offer multiple benefits. Early diagnosis, followed by appropriate treatment, leads to lower morbidity and improvement in lung function, cognitive development, nutrition, and overall growth [2].

In 1979, NBS for CF was made possible through the measurement of immunoreactive trypsinogen (IRT) using a dried blood spot specimen [3]. A second IRT measurement, known as the IRT-IRT technique, was conducted for infants whose first IRT levels were elevated or out of range [4, 5].

In the 1990s, following the identification of the CFTR gene and the c.1521_1523del variant, some NBS programs adopted the IRT-DNA protocol for CF NBS [6-8]. This procedure improved the sensitivity of screening and allowed for the rapid identification of infants with CF while lowering the IRT cutoff. However, diagnoses became biased toward infants of European ancestry, who were more likely to have the F508del mutation. Over the next 20 years, breakthroughs in genetic testing technology, the detection of more CFTR variants, and a greater understanding of variant pathogenicity and genotype-phenotype links have led to the development of extended multivariant panel analysis, which has become the predominant IRT-DNA technique [9].

Since 2010, all US states have mandated NBS for CF, which is overseen by state public health departments [10]. State-specific CF NBS algorithms and follow-up protocols can affect equity, sensitivity, and timeliness of interventions. In Saudi Arabia, Prince Sultan Military Medical City (PSMMC) in Riyadh is the only health institution where CF NBS is performed.

Implementing CF NBS improves nutritional outcomes and lung function in infants, even after accounting for advancements in clinical care [11, 12]. Compared with infants whose condition is detected early, infants whose symptoms are not detected through NBS or who have delayed presentation to the specialist exhibit worse clinical outcomes [11, 13, 14]. Sweat testing, clinical examination, or hospitalization at an earlier age in a CF center is correlated with better results, including greater development [13].

Regarding the global incidence of CF, in European populations, the incidence of CF is estimated to be one in 2,500 live births [14]. However, recent data from NBS programs indicate a lower incidence of CF than that in previous years, which was later estimated to be between one in 3,000 and one in 6,000 [15, 16]. In Europe, the incidence of CF varies from one in 1,353 in Ireland [17] to one in 25,000 in Finland [18], with an average of one in 4,500 in Western Europe [19-21] and one in 6,000 in Northern and Central Europe [22-24]. In Australasia, where an NBS program has been implemented for a long time, the incidence is well-documented, with an average of one in 3000 [25]. The incidence is around one in 3,300 in Canada [26] and one in 4,000 in the United States, with significant variations across ethnicities [27, 28]. Although CF is more widely recognized in Asian populations, its prevalence is still underestimated in many nations. Furthermore, the prevalence of consanguinity is higher in the Middle East than in East Asia. The incidence of CF varies from one in 2,560 in Jordan [29] to one in 350,000 in Japan [30], and the incidence has been predicted to be between one in 10,000 and one in 100,000 in India [31, 32]. Limited data are available for African populations [33].

Data concerning NBS for and the incidence of CF in Saudi Arabia are scarce. The objective of our study is to evaluate the primary outcome of identifying confirmed CF cases using a single-center IRT-IRT NBS protocol prior to multi-center expansion while assessing secondary outcomes, including diagnostic performance indices, optimal IRT cutoff thresholds, regional incidence, and CFTR genotype distribution.

## Materials and methods

This retrospective study included newborns who underwent CF screening at PSMMC, Riyadh, Saudi Arabia, between May 2017 and April 2019. During the study period, 10,286 newborns underwent CF screening using an IRT-IRT approach, with IRT measured at the initial screening and again on Day 5. Infants who underwent further diagnostic evaluation were assessed using sweat chloride testing. During the study period, 23 infants underwent sweat chloride testing, of whom three were classified as confirmed CF cases based on the diagnostic evaluation. Genetic testing was subsequently performed in the confirmed CF cases. Demographic and laboratory data, including Day 1 and Day 5 IRT concentrations, sweat chloride results, and available genetic testing results, were obtained from relevant laboratory, clinical, and CF clinic records. The total screened cohort (N = 10,286) was used to estimate the incidence of CF, whereas diagnostic and comparative analyses were performed according to the availability of the relevant laboratory and diagnostic measurements. The distinction between the total screened cohort, infants undergoing sweat chloride testing, and confirmed CF cases was maintained throughout the analyses.

The newborn screening protocol for CF depends on measuring the concentration of IRT in dried blood spot specimens via a two-tiered IRT sequence: an initial heel-prick dried blood spot test on Day 1 post birth, followed by a repeat IRT test on Day 5 for infants with elevated initial levels. The dried blood spot specimens were collected from newborns on Guthrie cards. IRT concentrations were measured using a commercially available assay (Neonatal IRT Kit, Perkin Elmer, Finland). Briefly, 0.3-inch (3.2 mm) dried blood spot discs, along with a set of calibrators and quality control (QC) samples, were punched into 96-well microplates using an automatic puncher (Panthera, Turku, Finland). The plates were then loaded into the Genetic Screening Processor (GSP, Perkin Elmer, Turku, Finland). The assay utilizes a direct sandwich time-resolved fluoroimmunoassay technique, where two monoclonal antibodies bind to distinct antigenic determinants on the IRT molecule, producing a fluorescence signal proportional to the IRT concentration. A cutoff value of 60 ng/mL was used to differentiate between positive and negative results. The validated limit of detection (analytical sensitivity) was 2.2 ng/mL. For each 96-well plate, a six-point calibration curve (0, 25, 50, 100, 250, and 500 ng/mL) was processed. QC samples at three levels (30, 70, and 110 ng/mL) were included at the beginning and end of every analytical run. All QC results were strictly evaluated and verified prior to reporting patient findings; any run with unacceptable QC parameters was repeated in its entirety.

Infants with persistently high IRT concentrations were referred to the pediatric CF clinic for diagnostic confirmation using quantitative Nanoduct® Neonatal Sweat Analysis System (Model 1030; ELITechGroup Inc., Logan, UT, USA), categorized as normal (<30 mmol/L), intermediate (30-79 mmol/L), or positive (≥80 mmol/L), followed by CFTR gene mutation analysis. Infants presenting with multiple congenital anomalies (predominantly gastrointestinal or renal) and those whose families were unresponsive to communications for confirmatory testing were excluded from the study cohort.

Notably, our protocol employed a lower threshold for normal values (<30 mmol/L) than the standard threshold of 50 mmol/L typically utilized in screenings. This ultraconservative approach deliberately created a cohort with broader intermediate levels (30-79 mmol/L) as a rigorous safety net to ensure that no cases were missed, while maintaining high specificity through a definitive positive cutoff at ≥80 mmol/L [34-35]. Medical charts of the included patients were reviewed using a standardized data collection sheet validated by the study authors to extract demographic data, IRT values, sweat chloride concentrations, and genetic study results

Ethical consideration

This study was conducted after ethical approval was obtained from the Conjoint Health Research Ethics Board of PSMMC (reference number: HP-01-R079 ID: 1622). Patient confidentiality was maintained throughout the study by assigning each patient a unique identification number, and the collected data were maintained in a password-protected database.

Statistical analysis

Statistical analysis was performed using IBM SPSS Statistics for Windows, version 26.0 (IBM Corp., Armonk, NY, USA). Continuous variables were assessed for normality using the Kolmogorov-Smirnov test and are presented as mean ± standard deviation (SD) or median with interquartile range (IQR), as appropriate. Categorical variables are presented as frequencies and percentages. The diagnostic performance of Day 5 IRT for identifying confirmed CF cases was evaluated using receiver operating characteristic (ROC) curve analysis, with Day 5 IRT as the test variable and confirmed CF status as the outcome. The ROC analysis included the three confirmed CF cases as positive observations. The optimal cutoff was determined using both the Youden Index and the shortest distance to the top-left corner of the ROC curve. Sensitivity, specificity, positive predictive value (PPV), and negative predictive value (NPV), together with their 95% confidence intervals, were calculated, and the area under the ROC curve (AUC) was reported with its 95% confidence interval. Cross-validation and bootstrapping were not performed because of the very small number of confirmed CF cases; therefore, the ROC-derived estimates were considered exploratory and interpreted cautiously. Comparisons of Day 5 IRT levels between confirmed CF and non-CF infants were performed using the Mann-Whitney U test. Paired comparisons between Day 1 and Day 5 IRT levels were performed using the Wilcoxon signed-rank test. The association between Day 5 IRT and sweat chloride concentration was assessed using Pearson’s correlation coefficient. The incidence proportion was calculated as the number of confirmed CF cases divided by the total number of screened newborns, with exact binomial 95% confidence intervals. All statistical tests were two-tailed, and p < 0.05 was considered statistically significant.

## Results

Between May 2017 and April 2019, 10,286 newborns underwent CF screening at PSMMC, Riyadh, Saudi Arabia. The baseline biochemical characteristics of the included sample are summarized in Table 1. The mean (±SD) value of Day 5 IRT levels was 19.47 ng/mL (±13.23 ng/mL), and the mean (±SD) value of the sweat chloride concentration was 58.09 mmol/L (±34.07 mmol/L). The results revealed three confirmed CF cases (sweat chloride test >80 mmol/L), accounting for 0.029% of the total cases.

**Table 1:** Baseline characteristics of screen-positive newborns (n = 10286) *Among those who underwent sweat testing. Data are represented as mean ± standard deviation (SD) for continuous, normally distributed variables, median with interquartile range (IQR) for skewed variables, absolute range (Min–Max), and frequencies with percentages (%) for categorical variables. IRT: immunoreactive trypsinogen; CF: cystic fibrosis

	Mean ± SD	Median (IQR)	Min–Max
Day 5 IRT (ng/mL)	19.47 ± 13.23	16.70(12.00-23.30)	5 - 500
Sweat chloride (mmol/L)*	58.09 ± 34.07	47.00(38.00-59.00)	27-162
Confirmed CF cases (sweat chloride test > 80), n (%)	3(0.029)		
Number of mutations detected, n (%)	3(0.029)		

Sweat chloride tests were conducted for 23 infants who were identified with high IRT levels and followed up at our clinic, and their categorizations are shown in Table 2. One patient (4.3%) had normal levels (<30 mmol/L; normal), 18 (78.3%) had intermediate levels (30-79 mmol/L), and four (17.4%) exhibited positive results (≥80 mmol/L). Of these four patients, three underwent genetic testing, and the results are shown in Table 3. One patient (33.33%) was positive for the homozygous c1416delG gene, another patient (33.33%) was positive for the homozygous I1234V gene, and the third patient was positive for the homozygous c1418delG gene ، while the fourth patient was lost to follow-up due to non-adherence to scheduled appointments, precluding genetic confirmation.

**Table 2:** Distribution of sweat chloride results (N = 23 tested) Data are represented as absolute counts (N) and relative percentages (%).

Sweat chloride category	N	%
< 30 mmol/L (Normal)	1	4.3
30–79 mmol/L (Intermediate)	18	78.3
≥ 80 mmol/L (Positive)	4	17.4

**Table 3:** Genetic testing results among sweat-positive infants (N=3) Data are represented as absolute counts (N) and relative percentages (%).

Gene	N	%
Homozygous c1416delG	1	33.33
HomozygousI1234V	1	33.33
Homozygous c1418delG	1	33.33

The incidence of CF diagnosed through NBS during the study period is provided in Table 4. Three infants were diagnosed with CF, corresponding to an incidence of 2.9 in 10,000 live births and an incidence ratio of 1:3429. The results revealed that the mean (±SD) value of Day 5 IRT levels was significantly higher in infants with confirmed CF than in infants without CF (136.07 ± 27.24 vs. 19.44 ± 13.08 ng/mL, respectively; p = 0.003), as shown in Table 5.

**Table 4:** Incidence of cystic fibrosis (CF) diagnosed via newborn screening (2017–2019) Data are represented as total counts, calculated incidence rate per 10,000 live births, and the absolute incidence ratio.

Total newborns screened	Confirmed CF cases	Incidence per 10,000	Incidence ratio
10286	3	2.9	1 : 3429

**Table 5:** Comparison of Day 5 IRT between confirmed CF and non-CF infants Data are represented as mean ± standard deviation (SD) and median with interquartile range (IQR). Statistical comparison was performed using the non-parametric Mann-Whitney U test. A p-value of <0.05 is considered statistically significant (∗ highly significant at p=0.003). Mann-Whitney Test was used. IRT: immunoreactive trypsinogen; CF: cystic fibrosis

	CF (n=3)		Non-CF (n=10283)		Test statistic	p-value
	(Mean ± SD)	Median (IQR)	(Mean ± SD)	Median (IQR)	Mann-Whitney U = 34.00	
Day 5 IRT (Mean ± SD)	136.07 ± 27.24	127.80(113.40 - - )	19.44 ± 13.08	16.70(12.00 - 23.30)		0.003*

**Figure 1:**
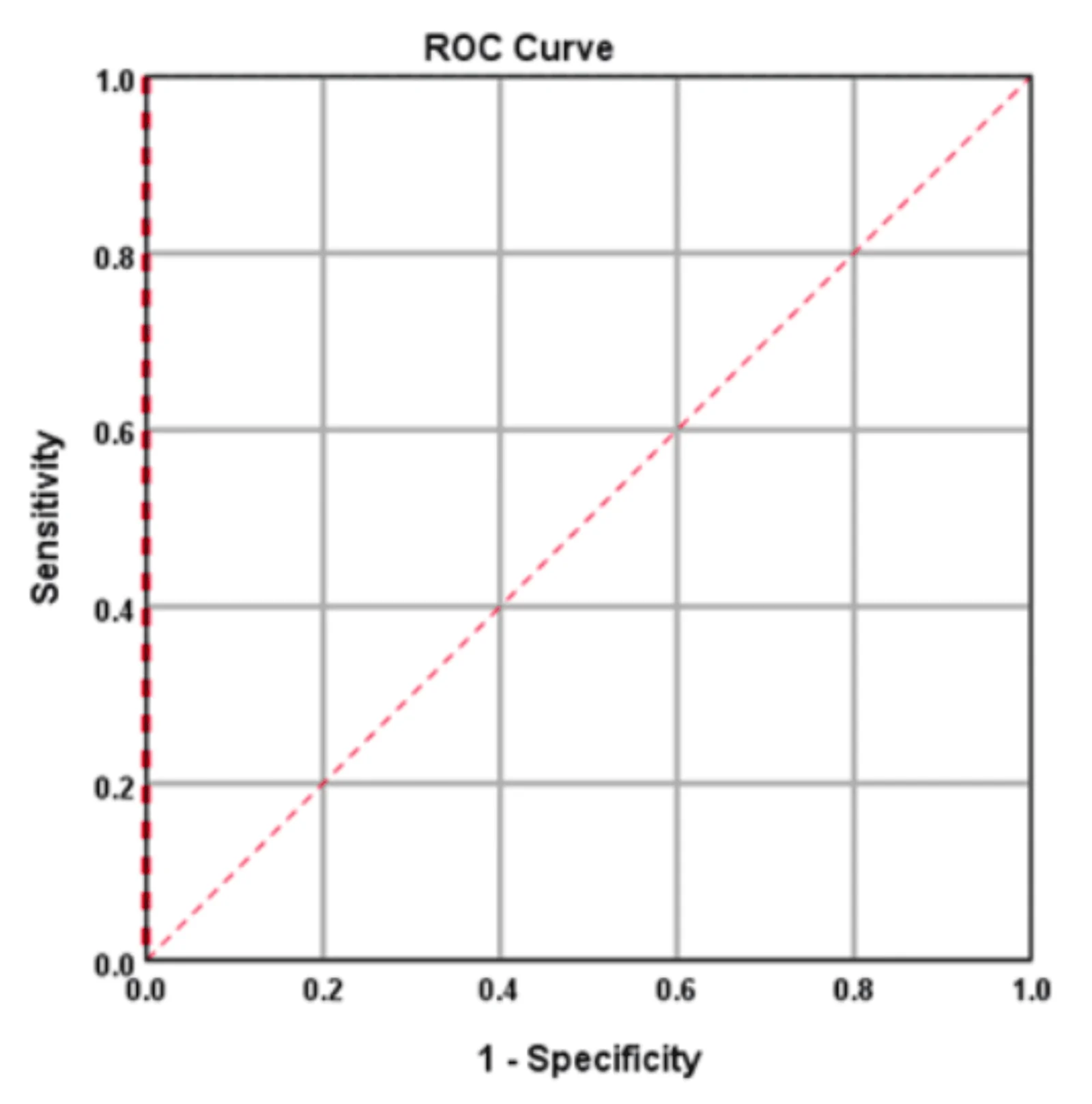
ROC curve analysis for IRT cutoff IRT: immunoreactive trypsinogen; ROC: receiver operating characteristic

ROC curve analysis demonstrated the excellent diagnostic discrimination ability of IRT levels. The optimal cutoff value for IRT levels was 114.9 ng/mL, which was determined using both the Youden index and the shortest distance method. At this threshold, sensitivity was 75.0% (95% CI: 19.4-99.4), and specificity was 99.8% (95% CI: 99.6-99.9). The PPV was 12.5% (95% CI: 2.7-32.4), and NPV was 99.99% (95% CI: 99.95-100). The AUC remained extremely high at 0.999 (95% CI: 0.998-1.000; p = 0.001), indicating the outstanding overall performance of the IRT screening test (Table 6, Figure 1).

**Table 6:** ROC curve analysis for IRT cutoff Parameters are presented as calculated threshold values accompanied by their 95% confidence intervals (CI). The p-value reflects the statistical significance of the area under the curve (AUC) relative to a null baseline (AUC = 0.5), where p<0.05 indicates a statistically significant diagnostic performance. IRT: immunoreactive trypsinogen; PPV: positive predictive value; NPV: negative predictive value; ROC: receiver operating characteristic

Method	Optimal cutoff (ng/mL)	Sensitivity % (95% CI)	Specificity % (95% CI)	PPV % (95% CI)	NPV % (95% CI)	AUC (95% CI)
Youden Index	114.9	75.0 (19.4–99.4)	99.8 (99.6–99.9)	12.5 (2.7–32.4)	99.99 (99.95–100)	0.999 (0.998–1.000)
Short Distance	114.9	75.0 (19.4–99.4)	99.8 (99.6–99.9)	12.5 (2.7–32.4)	99.99 (99.95–100)	0.999 (0.998–1.000)

Among the 23 infants who underwent sweat chloride testing, a strong positive correlation was observed between Day 5 IRT levels and sweat chloride concentration (r = 0.698, p < 0.001), as shown in Table 7. As indicated in Table 8, among 536 infants who underwent paired measurements, the mean (±SD) value of Day 1 IRT levels was significantly higher than that of Day 5 IRT levels (22.53 ± 14.10 vs. 17.29 ± 11.01 ng/mL; p < 0.001), with a mean paired difference of 5.27 ng/mL.

**Table 7:** Correlation between IRT and sweat chloride Data represent the numerical test statistic calculated via the Pearson correlation coefficient (r). **Statistical significance threshold is defined at p<0.01(two-tailed) ( highly significant at p<0.001). IRT: immunoreactive trypsinogen

Variable	r	p-value
IRT day 5 vs Sweat chloride	0.698^**^	<0.001

**Table 8:** Difference between Day 1 and Day 5 IRT   (N=536) Data values are represented as mean ± standard deviation (SD), mean paired difference, median with interquartile range (IQR), and absolute range (Min–Max). Statistical evaluation of paired samples was performed via the non-parametric Wilcoxon signed-rank test. A p-value of <0.05 is considered statistically significant (∗ highly significant at p<0.001). IRT: immunoreactive trypsinogen

	Mean ± SD	Mean difference	Median (IQR)	Min–Max	Test statistic	p-value
Day 1 IRT (ng/mL)	22.53 ± 14.10	5.27	18.70(14.03 - 27.08)	5 - 172	Wilcoxon signed-rank Z = -12.81	< 0.001*
Day 5 IRT (ng/mL)	17.29 ± 11.01		14.90(10.80 - 20.60)	5 - 103.90		

## Discussion

This retrospective study provides a critical evaluation of NBS outcomes for CF at PSMMC. Our findings demonstrate a CF incidence of 2.9 in 10,000 live births (1:3429) in our screened cohort. This rate is notably higher than previously reported Saudi national averages, including the ratio of 1:4243 cited in a previous study [36]. This discrepancy indicates the vital importance of obtaining high-volume, center-specific data to capture the true disease burden within a population where genetic risk is elevated by high consanguinity rates. However, compared with the latest global estimates [16], the incidence of CF in Saudi Arabia is almost within the range of one in 3,429 live births vs. one in 3,000 and one in 6,000 live births globally. Additionally, the incidence rate of CF in Saudi Arabia is consistent with that reported in some countries in the Middle East and among individuals of Caucasian ancestry [36]. The incidence of CF in the Arab world varies from one in 2,500 to one in 16,000 live births. According to reports, the incidence is one in 1680 in Morocco, one in 2410 in Oman, one in 2560 in Jordan, one in 2664 in Egypt, one in 3500 in Kuwait, and one in 15,876 in the United Arab Emirates [37]. Moreover, 82 instances were found in Qatar [38]

Globally, in most regions, the prevalence of CF seems to be decreasing. However, the incidence ratio in the current pilot study is higher than that in a previous report in Saudi Arabia at 1:4243 [36]. 

The identification of the CFTR gene in 1989 has led to a better understanding of the etiology of this disease. The identification of the gene has significantly improved the diagnosis and treatment of CF patients, with benefits to their families, and investigations of genotype/phenotype associations have provided a deeper understanding of phenotypic variability.

Early nutritional status and growth outcomes in newborns with CF are significantly influenced by prompt treatment initiation after a positive result from NBS. According to recent data from a US registry-based study, infants who started receiving CF care earlier (median, 10 days) exhibited significantly better weight-for-age and length-for-age z scores at both the first clinical visit and at one year of age than those whose care started later (median, 47 days) did [12]. These results underscore the importance of minimizing delays between diagnosis and therapy because even slight variations in timing can have quantifiable and long-lasting effects on early childhood growth trajectories.

The observed institutional detection rate of 1:3429 in this single-center cohort provides initial baseline data. However, several limitations of this study warrant consideration. First, as a single-center retrospective study conducted at PSMMC, our findings may not fully capture regional variations across the entire Kingdom of Saudi Arabia. Second, while our overall screening cohort was large (N=10,286), the absolute number of infants undergoing diagnostic sweat testing (n=23) and confirmed CF cases (n=3) was small. Third, diagnostic sweat evaluation was conducted using sweat conductivity (Nanoduct®) rather than direct quantitative sweat chloride measurement, requiring cautious comparison with studies utilizing classical iontophoresis. Finally, one infant with elevated conductivity was lost to follow-up prior to confirmatory genetic testing. Future multi-center prospective studies involving larger confirmatory testing cohorts are needed to validate these preliminary findings

## Conclusions

Although the IRT-IRT protocol worked well for detecting CF early, the high number of intermediate sweat test results remains a challenge, particularly alongside high regional rates of consanguinity. While this pilot study shows that screening is feasible, expanding it nationwide will require larger multicenter studies to build a reliable national program.
